# Translation and validation of the artificial intelligence anxiety scale in German

**DOI:** 10.1371/journal.pone.0333073

**Published:** 2025-10-08

**Authors:** André Hajek, Larissa Zwar, Ariana Neumann, Razak M. Gyasi, Dong Keon Yon, Supa Pengpid, Karl Peltzer, Hans-Helmut König

**Affiliations:** 1 Department of Health Economics and Health Services Research, University Medical Center Hamburg-Eppendorf, Hamburg Center for Health Economics, Hamburg, Germany; 2 Department of Psychology, Brock University, St. Catharines, Ontario, Canada; 3 African Population and Health Research Center, Nairobi, Kenya; 4 National Centre for Naturopathic Medicine, Faculty of Health, Southern Cross University, Lismore, New South Wales, Australia; 5 Center for Digital Health, Medical Science Research Institute, Kyung Hee University College of Medicine, Seoul, South Korea; 6 Department of Health Education and Behavioral Sciences, Faculty of Public Health, Mahidol University, Bangkok, Thailand; 7 Department of Public Health, Sefako Makgatho Health Sciences University, Pretoria, South Africa; 8 Department of Healthcare Administration, College of Medical and Health Science, Asia University, Taichung, Taiwan; 9 Department of Psychology, University of the Free State, Bloemfontein, South Africa; 10 Department of Psychology, College of Medical and Health Science, Asia University, Taichung, Taiwan; University of Potsdam: Universitat Potsdam, GERMANY

## Abstract

**Aim:**

Artificial intelligence anxiety refers to fear due to challenges caused by AI-related changes in one’s own life. As the first study, our aim was to translate and validate the German version of the Artificial Intelligence Anxiety Scale (AIAS-G). Furthermore, norm values (i.e., reference scores derived from the population) were presented.

**Methods:**

Data were used from a quota-based sample of the adult population in Germany spanning from 18 to 74 years (n = 3,270) reflecting the German population in terms of sex, age and federal state. Data were collected in January 2025. The translation process followed established guidelines. Reliability was determined (in terms of Cronbach’s alpha and McDonald’s omega). Confirmatory factor analysis was conducted to examine construct validity. Concurrent validity was investigated by calculating pairwise correlations of AIAS-G with depressive symptoms, anxiety symptoms, life satisfaction and ikigai (Japanese concept mainly referring to meaning/purpose in life). Moreover, norm values were offered (also for specific sociodemographic groups). The AIAS-G sum score ranges from 21 to 147, with higher values corresponding to a higher AI anxiety level.

**Results:**

Cronbach’s alpha was .97 for the AIAS-G (subscales from .94 to .98). The mean AI anxiety level was 69.6 (SD: 32.6), with highest mean levels among women, older adults, individuals being divorced/widowed, individuals with low education, and retired individuals. The four-factor model originally proposed was substantiated by the findings of the confirmatory factor analysis. Higher levels of AI-related anxiety were associated with more depressive symptoms (r = .32, p < .001), more anxiety symptoms (r = .34, p < .001), lower life satisfaction (r = −.16, p < .001) and lower ikigai levels (r = −.21, p < .001).

**Conclusion:**

The AIAS-G is a psychometrically sound instrument designed to determine AI anxiety levels among German speakers. Further translation and validation studies are necessary to enable comparisons across various countries.

## 1 Introduction

Particularly in the past years, artificial intelligence (AI) has markedly gained popularity. AI tools such as ChatGPT or Gemini are nowadays widely used globally. AI is increasingly being used across a variety of sectors, including healthcare, finance, retail, information technology, and the automotive industry. Individuals may perceive AI as opportunities (e.g., to make processes markedly more efficient) but also as risks (e.g., AI may replace their jobs in the future). Therefore, in a broad sense, they may have positive or negative attitudes towards AI (e.g., they may accept AI or have concerns) [1–4]. Thus, it is plausible that individuals may develop AI-related anxiety. AI-related anxiety (or: AI anxiety) refers to the fear due to challenges caused by AI-related changes in one’s own life [5].

Only few studies exist dealing with the topic of AI-related anxiety (e.g., [6]). Most of the existing studies focused on quite specific associations and are based on small, selective samples (mainly) from Turkey. For example, one study showed a negative association between general self-efficacy and AI-related anxiety based on 317 nursing students (one public nursing school in Turkey) [7]. A further study [8] showed that AI-related anxiety mediated the relationship between AI literacy and attitudes toward AI (also based on student nurses in eastern Turkey). Another Turkish study [9] revealed a positive association between AI-related anxiety and unemployment anxiety level among university students. AI-learning anxiety can also predict attitudes toward AI among younger to middle-aged individuals in Turkey using data from a convenience sample [10].

Thus far, tools to quantify AI-related anxiety are almost missing. For example, the 5-item attitudes towards artificial intelligence (ATAI) [1] exists. However, the ATAI refers to AI attitudes in general and the fear subscale only covers three items. More precisely, only the Artificial Intelligence Anxiety Scale (AIAS) exists to specifically and comprehensively quantify AI-related anxiety levels. This tool was developed and validated in English by Wang and Wang [5]. It encompasses four dimensions: (1) learning (referring to the fear of learning AI technologies), (2) job replacement (referring to the fear of negative consequences of AI on working life), (3) sociotechnical blindness (referring to the fear resulting from a limited understanding of how AI depends on humans; expressing fear that humans will be sidelined by the introduction of AI), and (4) AI configuration (referring to the fear of humanoid AI). In this sense, AI is not simply “lines of code that perform computational operations” (p. 2268) [11]. Such “AI programs have to be put into physical computers that perform operations that have meaning for humans. Consider a furnace that is turned on by signals triggered by a digital output, an airplane that changes direction when software operates, a bid for a particular stock by a software agent for stock market trading.“ (p. 2268) [11].

Wang and Wang [5] provided details of the AIAS’s theoretical foundation of the AIAS. In short [5]: The theoretical basis of the AIAS lies in the research of technophobia and anxiety relating to advanced technologies, particularly AI. Technophobia involves irrational fears and negative attitudes towards technology [12]; anxiety is a key construct in this context because it reflects emotional responses that can influence behaviour. Anxiety can be trait-like (stable) or state-like (transient) [13]. Technological anxiety can be commonly considered as state anxiety (e.g., [14]). The AIAS [5] uses concepts related to AI-anxiety such as computer (e.g., multidimensional computer anxiety rating scale [15]) and robot anxiety (e.g., multidimensional robot anxiety scale [16]). However, unlike such aforementioned related anxieties, AI-related anxiety may stem from confusion about autonomy, sociotechnical blindness and inaccurate perceptions of technological development [11]. As defined earlier, AI-related anxiety can be understood as a multidimensional emotional response that can hinder interaction with AI [5]. Additional details are provided by Wang and Wang [5].

Recent studies translated and validated this tool into Persian [17] or Turkish [6] based on selective samples. Both studies confirmed the four-factor solution and demonstrated excellent reliability (.95 for the Persian version [17];.96 for the Turkish version [6]). However, a German version of the AIAS is missing thus far. Therefore, the aim of this present study was the translation and validation of a German version of the AIAS. Such knowledge is relevant since it eases future cross-country comparisons and improves our understanding of AI-related anxiety within the German population. Furthermore, it can increase our future understanding of the predictors and consequences of AI-related anxiety among populations speaking the German language. For example, upcoming research could examine the sociodemographic characteristics associated with AI-related anxiety based on the AIAS-G. This tool could also be used to examine how it predicts the motivation or willingness to use AI-based products or services (e.g., at the workplace or in the healthcare sector) [5]. Moreover, a better understanding of AI-related anxiety may inspire subsequent awareness campaigns (to encourage acceptance).

## 2 Materials and methods

### 2.1 Sample

For our current study, we used data from a large sample of individuals in Germany who were selected based on quotas (crossed quota: sex x age; uncrossed quota: federal state). Of note that quota sampling is a non-probability sampling technique that ensures specific representation of certain subgroups (listed above) in the sample by setting predetermined quotas for each subgroup [18]. A key strength is that data were collected in such a way to reflect the German adult population in terms of age, sex (crossed quota: sex x age), and geographical location (uncrossed quota: federal state). A key weakness is that quota sampling can introduce bias because it does not use random selection. More precisely, the aforementioned quotas regarding sex, age and federal state may be fulfilled, whereas we cannot dismiss the possibility that our respondents differ in other unobserved factors (e.g., interest in the specific topic) from the general adult population.

The age span ranged from 18 to 74 years (n = 3,270). The sample size was adequate (a priori power analysis with r = .3 (vs. null correlation), power of .8 and significance level of .05; estimated sample size = 85). Furthermore, according to a rule of thumb, a sample size of at least 10 times the number of items is recommended for a CFA [19]. Thus, the recommended size for a CFA (which would be 210 here) would be largely exceeded. More formally, we also computed the sample size for the CFA according to Kim [20] (expected CFI: .95, number of items: 8, 6, 4 and 3; average factor loading: .6, average factor correlation: .3, significance level = .05, power = .8; resulting estimated sample size: 300). Our present larger sample of n = 3,270 individuals also allows statements to be made for subgroups (norm values for the German version).

We therefore aimed for a sample of at least 3,000 individuals, ultimately resulting in a sample of n = 3,270 individuals because the market research company has generously rounded up here. In total, 28,886 invitations were sent. The resulting low response rate is comparable to the response rate of other recent online surveys focusing on the general adult population in Germany (e.g., [21,22]). Overall, 3,272 individuals completed the questionnaire. Thereof, two individuals were removed as part of quality control resulting in a sample of n = 3,270 individuals.

Furthermore, 221 individuals dropped out (i.e., those individuals completed only some parts of the questionnaire), 42 individuals were screened out (e.g., because they did not meet the inclusion criteria) and 418 individuals could not be considered because the quota were already fulfilled. Individuals completing the total questionnaire did not significantly differ from those individuals who only partially completed the questionnaire (e.g., in terms of sex (p = .28), age (p = .55), federal state (p = .76), employment status (p = .52) or self-rated health (p = .87)).

Data collection took place online in January 2025. The market research company Bilendi – an ISO certified company – was responsible for collecting the data. No pre-registration was made for our study. Bilendi offered a small remuneration (so-called mingle points). As soon as participants have collected enough points, they can have an amount transferred to them, exchange them for rewards or donate them.

This survey was a multi-topic one (with most questions related to well-being). On average, respondents took slightly less than 34 minutes to answer the questions.

Two inclusion criteria were applied: Individuals had to be between 18 and 74 years and residing in Germany. Individuals aged 17 and younger or individuals aged 75 or older and those individuals residing outside of Germany were therefore excluded. Further exclusion criteria were not applied.

Before starting the survey, all participants provided written informed consent by agreeing to the online consent form, which is a standard procedure for conducting online surveys. After giving their consent, respondents first answered key sociodemographic questions (e.g., age, gender, state, education, and employment status), followed by questions on the use of health services, pet-related stress (only for pet owners), health status (especially the presence of various chronic diseases and self-rated health), and lifestyle (especially smoking, drinking, exercise, and nutrition). This was followed by the AIAS questionnaire. After that, questions were asked about climate anger, mental health (depressive and anxiety symptoms), loneliness, isolation and withdrawal, ikigai and life satisfaction, and online gaming addiction (only for individuals playing such games).

Ethical approval for this study was provided by the Local Psychological Ethics Committee at the University Medical Center Hamburg-Eppendorf (LPEK-0849).

### 2.2 Translation process

Closely following well-known guidelines [23], the translation process was conducted. A renowned professional company (tolingo) was responsible for translation of the AIAS. The ISO-certified company Tolingo is an important translation agency operating internationally. Additional details can be found on their website (https://www.tolingo.com/). Both translators of tolingo were native German speakers. While one had prior knowledge of the research field, the second one had no prior knowledge in this field (i.e., “naive”). Both translators translated the AIAS independently into German language. Thereafter, two of the authors (AH, HHK) harmonized both translated versions. A psychologist (AN) supported this process. The subsequent back-translation of the harmonized version into English was performed independently by two English native speakers from tolingo. Again, while one was familiar with this research field, the second one had no prior knowledge in this research area (“naive”). Differences were resolved by means of discussions (AH, AN, HHK). The three authors mentioned (AH, HHK and AN) then checked the German version again very thoroughly (e.g., with regard to semantic, idiomatic and conceptual equivalence). The authors then jointly decided on a final version. In a small, subsequent quantitative pretest (with n = 34), we examined the response distribution for all 21 items. As the distribution for all 21 items was unremarkable (e.g., with regard to extreme values), no further changes were made. The German version of the AIAS (briefly: AIAS-G) is provided in Table 1, consisting of 21 items. The AIAS items were introduced as follows: “To what extent do you agree with the following statements?”. The answer categories range from 1 (strongly disagree) to 7 (strongly agree). By summarizing all 21 items (which were scored in the same direction), the final score was calculated. This final score ranges from 21 to 147, with higher values corresponding to a higher AI anxiety level.

**Table 1:** German version of the AIAS.

1. Ich habe Angst davor, alle speziellen Funktionen zu erlernen, die eine KI-Technologie/ein KI-Produkt mit sich bringt.
2. Ich habe Angst davor, zu lernen, wie man KI-Technologie/-Produkte verwendet.
3. Ich habe Angst davor, zu lernen, wie man bestimmte Funktionen einer KI-Technologie/eines KI-Produkts verwendet.
4. Ich habe Angst davor, zu lernen, wie eine KI-Technologie/ein KI-Produkt funktioniert.
5. Ich habe Angst davor, zu lernen, wie man mit einer KI-Technologie/einem KI-Produkt interagiert.
6. Ich habe Angst davor, an einem Kurs zur Entwicklung von KI-Technologien/-Produkten teilzunehmen.
7. Ich habe Angst davor, die Anleitung einer KI-Technologie/eines KI-Produkts zu lesen.
8. Ich habe Angst, nicht mit dem Fortschritt von KI-Technologien/-Produkten mithalten zu können.
9. Ich habe Angst, dass eine KI-Technologie/ein KI-Produkt uns abhängig machen könnte.
10. Ich habe Angst, dass eine KI-Technologie/ein KI-Produkt uns noch fauler machen könnte.
11. Ich habe Angst, dass eine KI-Technologie/ein KI-Produkt Menschen ersetzen könnte.
12. Ich habe Angst, dass eine weit verbreitete Nutzung menschenähnlicher Roboter Menschen ihre Arbeitsplätze wegnehmen könnte.
13. Ich habe Angst, dass ich im Falle der Nutzung von KI-Technologien/-Produkte abhängig von ihnen werde und einen Teil meiner Fähigkeiten im Bereich des logischen Denkens verliere.
14. Ich habe Angst, dass KI-Technologien/-Produkte Arbeitsplätze ersetzen könnten.
15. Ich habe Angst, dass eine KI-Technologie/ein KI-Produkt missbraucht werden könnte.
16. Ich habe Angst vor verschiedenen Problemen, die eine KI-Technologie/ein KI-Produkt möglicherweise mit sich bringen könnte.
17. Ich habe Angst, dass eine KI-Technologie/ein KI-Produkt außer Kontrolle geraten und eine Fehlfunktion haben könnte.
18. Ich habe Angst, dass eine KI-Technologie/ein KI-Produkt zur Verselbständigung von Robotern führen könnte.
19. Ich finde menschenähnliche KI-Technologie/-Produkte (z. B. menschenähnliche Roboter) unheimlich.
20. Ich finde menschenähnliche KI-Technologie/-Produkte (z. B. menschenähnliche Roboter) einschüchternd.
21. Ich weiß nicht, wieso, aber menschenähnliche KI-Technologie/-Produkte (z. B. menschenähnliche Roboter) machen mir Angst.
Antwortoptionen:
Jeweils von 1 = stimme überhaupt nicht zu bis 7 = stimme voll und ganz zu

Note: The first statement refers to item 1. The second statement refers to item 2 and so on.

### 2.3 Concurrent validity: Other measures

Pairwise correlations of the AIAS-G with depressive symptoms, anxiety symptoms, life satisfaction and ikigai (Japanese concept mainly referring to meaning/purpose in life) were calculated. Since AI anxiety is a specific form of anxiety, we aimed to examine the association of AI-related anxiety with general anxiety symptoms. Due to the strong association between anxiety symptoms and depressive symptoms [24], we also examined the association between AI-related anxiety and depressive symptoms. Because specific forms of anxiety are also associated with well-being/meaning in life outcomes [25,26], such associations were also investigated. However, in total, we cautiously assume that the AIAS-G is more strongly associated with depressive and anxiety symptoms than with life satisfaction and ikigai.

The Patient Health Questionnaire-9 (PHQ-9) was used to quantify depressive symptoms [27]. This valid and widely used tool has nine items. By summing up all nine items, the final score was generated. This score varies from 0 to 27, whereby higher values reflect more depressive symptoms. In our study, Cronbach’s alpha was .90 (McDonald’s omega = .90). The psychometrically sound and widely-applied Generalized Anxiety Disorder-7 (GAD-7) [28] was used to assess anxiety symptoms. Based on the sum of all seven items, a sum score was calculated. This varies from 0 to 21, with higher values reflecting more anxiety symptoms. Cronbach’s alpha was .92 (McDonald’s omega = .92) in our study. The valid and widely-used Satisfaction with Life Scale (SWLS) [29] which consists of five items was used to measure life satisfaction. By summarizing all items, the final score ranges from 5 to 35, with higher values corresponding to greater life satisfaction. Cronbach’s alpha equaled .90 (McDonald’s omega = .91) in this study. The Ikigai-9-G [30] was used to quantify ikigai [31]. Based on all 9 items, a sum score was created (from 9 to 45, with higher values corresponding to higher ikigai-levels). In this study, Cronbach’s alpha was .88 (McDonald’s omega = .88). It is worth noting that the German versions of such tools have been validated and show favorable psychometric properties [30,32–34].

### 2.4 Statistics

A confirmatory factor analysis (CFA, with maximum likelihood, ML) was performed to investigate the underlying four-factor structure of the tool examined. Kline recommended that at least three items should load on a factor [19]. This recommendation is met in our study.

Several fit indices were calculated to check the model adequacy including Chi² statistic, Root Mean Square Error of Approximation (RMSEA), Standardized Root Mean Square Residual (SRMR), the normed fit index (NFI), the Relative Fit Index (RNI), the Comparative Fit Index (CFI), and the Incremental Fit Index (IFI). It is worth noting that we presented several fit indices to provide a comprehensive evaluation of model fit. This can help ensure an accurate interpretation of model adequacy and reduce the risk of relying on a single index (which may be potentially misleading).

A Satorra-Bentler-adjustment was employed to address non-normality [35] – due to the absence of multivariate normality (Doornik-Hansen test [36]: p < .001). Current recommendations were followed regarding the criteria for good measurement characteristics [37].

Pearson correlations of AIAS-G with depressive symptoms, anxiety symptoms, life satisfaction and ikigai were calculated to evaluate concurrent validity. They were classified as follows: small (r = .10 to .29), moderate (.30 to .49), large (.50 to .69), very large (.70 to .89), or extremely large (>.90) [38].

Cronbach’s alpha and McDonald’s omega were used to assess the reliability of the AIAS-G. A satisfactory and strong internal consistency was defined by values of 0.7 or higher or 0.8 or higher, respectively. Values of 0.9 or higher can be interpreted as excellent internal consistency [39].

Missing values were not present. Therefore, missing data techniques (e.g., multiple imputation) were not applied. In principle, previous studies have not yet suggested how to deal with missing values in the AIAS (and its translations into other languages). When responses are missing completely at random (MCAR) or missing at random (MAR), it is suggested to use appropriate techniques such as multiple imputation or full-information maximum likelihood to deal with missings in the AIAS (and its translations such as the AIAS-G). Further guidance is provided by Newman [40].

StataNow 18.5 (MP-Parallel Edition, Stata Corp., College Station, Texas) was used for data analysis. The user-written Stata commands “validscale” [41] and “omegacoef” [42] were employed. While the former one encompasses the statistical procedures to validate a tool, the second one computes McDonald’s omega.

## 3 Results

### 3.1 Characteristics of the sample and mean levels of AI anxiety

The mean age of the participants was 47.0 years (SD: 15.3 years) old and 50.4% were female (see Table 2). Overall, 47.5% of the individuals had a medium education (according to the ISCED-classification [43]). It is worth noting that our sample closely matched the target population in terms of sex x age, and federal state (S1 Table and S2 Table). This also means that sampling weights were not required.

**Table 2:** Norm values for AIAS (additionally stratified by sociodemographic factors).

Variables	N (%)	AIAS: Mean (SD)	p-value
Total sample	3,270 (100%)	69.6 (32.6)	
**Sex**			<.001
Male	1614 (49.4%)	65.5 (32.3)	
Female	1647 (50.4%)	73.6 (32.4)	
Diverse	9 (0.3%)	66.8 (19.0)	
**Age group**			.001
18 to 29 years	582 (17.8%)	70.1 (29.6)	
30 to 39 years	602 (18.4%)	66.6 (31.6)	
40 to 49 years	553 (16.9%)	66.5 (30.6)	
50 to 59 years	721 (22.0%)	70.7 (34.8)	
60 years and older	812 (24.8%)	72.6 (34.3)	
**Marital status**			<.001
Single	908 (27.8%)	68.6 (31.3)	
Divorced	277 (8.5%)	76.9 (34.2)	
Widowed	103 (3.1%)	76.7 (35.7)	
Living together: Married/Partnership	1867 (57.1%)	68.7 (32.6)	
Living separated: Married/Partnership	115 (3.5%)	67.8 (33.5)	
**Education (ISCED-classification)**			<.001
Low	337 (10.3%)	76.9 (32.8)	
Medium	1552 (47.5%)	71.6 (33.3)	
High	1381 (42.2%)	65.5 (31.2)	
**Migration background**			.33
No	2864 (87.6%)	69.8 (32.8)	
Yes	406 (12.4%)	68.1 (31.3)	
**Employment status**			<.001
Full-time employed	1629 (49.8%)	65.9 (31.6)	
Retired	649 (19.8%)	75.0 (34.6)	
Other	992 (30.3%)	72.1 (32.2)	

Notes: P-values are based on the t-statistic of the independent t-tests (used when comparing the means of two groups, in our study: migration background) or the F-statistic of one-way ANOVAs (used when comparing the means of three or more groups, in our study: all other variables) were conducted.

The mean level of AI anxiety was 69.6 (SD: 32.6; median: 70; 10% percentile: 22; 90% percentile: 112) among the total sample. In this section, norm values for key socio-demographic variables that are frequently included in questionnaires were also reported. There were significant differences between sociodemographic groups (in detail: see S3 Table). For example, while the mean level of AI anxiety was 65.5 (SD: 32.3) among men, it was 73.6 (SD: 32.4) among women. Individuals aged 40–49 years had a mean level of AI anxiety of 66.5 (SD: 30.6), whereas individuals aged 60–74 years had a mean level of AI anxiety of 72.6 (SD: 34.3). Divorced (76.9, SD: 34.2) or widowed individuals (76.7, SD: 35.7) also reported high mean levels of AI anxiety. Moreover, individuals with low education (76.9, SD: 32.8) and individuals being retired (75.0, SD: 34.6) also had comparably high mean levels of AI anxiety. In S4 Fig. and S5 Table, we report a histogram of subscale-scores and a detailed item-description, respectively.

### 3.2 Reliability

Table 3 depicted the internal consistency (in terms of Cronbach’s alpha and McDonald’s omega). Both, Cronbach’s alpha and McDonald’s omega for the AIAS-G equaled .97. Cronbach’s alpha and McDonald’s omega were also the same for the four subscales: .98 for the subscale learning, .94 for both the subscale job replacement and sociotechnical blindness, and.95 for the subscale AI configuration.

**Table 3:** Internal consistency for AIAS-G and the four subscales.

	AIAS-G	Subscale: learning	Subscale: Job replacement	Subscale: Sociotechnical blindness	Subscale: AI configuration
Cronbach’s alpha	.97	.98	.94	.94	.95
McDonald’s omega	.97	.98	.94	.94	.95

### 3.3 Validity

#### 3.3.1 Construct validity of the AIAS-G.

CFA was conducted to confirm the four-factor solution initially proposed for the AIAS. The results of the CFA endorse the model proposed by Wang and Wang [5]. Good to very good model fit in this sample was shown (e.g., RMSEA = .063, SRMR = .051, CFI = .955). More details are presented in Table 4. It is worth noting that the model fit of a one-factor solution was markedly poorer (Chi² (df: 189): 17888.77, RMSEA:.169, SRMR:.118, NFI:.666, RNI:.666, CFI:.666, IFI:.666).

**Table 4:** Confirmatory factor analysis (four-factor solution).

Chi²	df	RMSEA	SRMR	NFI	RNI	CFI	IFI
2592.61 (p < .001)	183	0.063	0.051	0.951	0.955	0.955	0.955

Notes: Satorra-Bentler adjusted goodness-of-fit indices were calculated.

The standardized factor loadings for the four-factor structure were shown in Table 5. Additionally, Pearson correlation between the subscales are also presented in Table 5. Of note that the standardized factor loadings frequently varied from about .85 to .95. Moderate to high positive associations were identified between the four subscales. Further details are presented in Table 5.

**Table 5:** Standardized factor loadings (SE in parentheses) of the AIAS-G and pairwise correlations (Pearson’s r) between the three subscales.

Items	Learning subscale	Job replacement subscale	Sociotechnical blindness subscale	AI configuration subscale
Item 1	0.89 (0.01)			
Item 2	0.95 (0.00)			
Item 3	0.96 (0.00)			
Item 4	0.96 (0.00)			
Item 5	0.95 (0.00)			
Item 6	0.89 (0.01)			
Item 7	0.90 (0.01)			
Item 8	0.81 (0.01)			
Item 9		0.85 (0.01)		
Item 10		0.84 (0.01)		
Item 11		0.90 (0.00)		
Item 12		0.88 (0.01)		
Item 13		0.79 (0.01)		
Item 14		0.90 (0.00)		
Item 15			0.85 (0.01)	
Item 16			0.91 (0.00)	
Item 17			0.93 (0.00)	
Item 18			0.89 (0.00)	
Item 19				0.92 (0.00)
Item 20				0.94 (0.00)
Item 21				0.93 (0.00)
Subscale: Learning	1			
Subscale: Job replacement	0.67	1		
Subscale: Sociotechnical blindness	0.57	0.86	1	
Subscale: AI configuration	0.65	0.79	0.80	1

Notes: all items were highly significant (p < .001).

#### 3.3.2 Concurrent validity of the AIAS-G.

Findings of pairwise correlations of AI-related anxiety levels (AIAS-G) with depressive symptoms, anxiety symptoms, life satisfaction and ikigai are shown in Table 6. Higher levels of AI-related anxiety were associated with more depressive symptoms (r = .32, p < .001), more anxiety symptoms (r = .34, p < .001), lower life satisfaction (r = −.16, p < .001) and lower ikigai levels (r = −.21, p < .001). More details are shown in Table 6.

**Table 6:** Pairwise correlations: concurrent validity analysis with n = 3,270.

Variables	1	2	3	4	5
1: AI-related anxiety levels (AIAS-G)	1				
2: Depressive symptoms (PHQ-9)	.32***	1			
3: Anxiety symptoms (GAD-7)	.34***	.81***	1		
4: Life satisfaction (SWLS)	−.16***	−.45***	−.43***	1	
5: Ikigai (Ikigai-9-G)	−.21***	−.40***	−.36***	.58***	1

Notes: Pearson correlations are displayed (bonferroni-adjusted significance levels are computed), ^*^ *p* < 0.05, ^**^ *p* < 0.01, ^***^ *p* < 0.001. AIAS-G: skewness: 0.26, kurtosis: 2.40; PHQ-9: skewness: 0.86, kurtosis: 3.24; GAD-7: skewness: 0.89, kurtosis: 3.11; SWLS: skewness: −0.36, kurtosis: 2.99; Ikigai-9-G: skewness: −0.45, kurtosis: 3.32.

## 4 Discussion

The aim of this present study was the translation and validation of the German AIAS version (AIAS-G) for the use in future research among populations speaking German. This current study is the first study translating and validating the German version of the AIAS. Moreover, norm values were presented. Consequently, this study increases our present knowledge in this area of research.

The four-dimensional structure which was originally proposed by Wang and Wang [5] was confirmed in our study by means of a confirmatory factor analysis. This is also in line with the Turkish and Persian version of the AIAS [6,17].

The original validation [5] also provided percentile and average scores for the AIAS. However, it should be emphasized at this point that there is currently no threshold value for possible pathological or problematic AI-related anxiety. There is also no established threshold for the 5-item ATAI. While the 10% percentile had a value of 58 (our study: 22), the median equaled 88 (our study: 70) and the 90% percentile was 130 (our study: 112). The mean of the AIAS was 91.4 (SD: 26.6), whereas it was 69.6 (SD: 32.6) in our study. The corresponding effect size (Cohen’s d – in absolute terms) for this mean difference was d = .68 – which is quite large. Such difference in AI-related anxiety may be mainly explained by cultural differences between Taiwan and Germany (e.g., fear of job losses due to AI because many people in Taiwan are directly affected by the production and use of new technology) and differences in the sociodemographic structure of the samples. For example, about 90% of the participants in the study by Wang and Wang [5] were 16–40 years old (and nearly 50% were aged 21–30 years), whereas less than 40% were up to 40 years in our study. Such younger Taiwanese individuals may more often fear that their current and future may be replaced by AI. This potential explanation is substantiated by the fact that 39.2% of the Taiwanese individuals replied with “yes” to the question whether the work content may be replaced by AI [5]. Comparing such mean levels is even more difficult compared to the Turkish study [6] because they solely focused on a very specific group of teachers (Persian study: college students [17]). We encourage upcoming research presenting norm values based on samples of the general adult population from other countries.

Beyond the aforementioned discussed factor of age group, significant bivariate differences in mean AIAS-G levels were identified depending on sex, marital status, education and employment status, whereas they did not differ depending on migration background. Higher mean AIAS-G levels among women correspond to previous research which often identified higher anxiety levels across several domains (dementia anxiety, war anxiety or climate anxiety [44–46]) in women compared to men in Germany. This may be attributed to the higher tendency to worry amongst women [46]. Differences according to marital status may be attributed to the fact that individuals talking to a partner may quickly discuss AI topics with their partner, whereas people who are widowed, for example, may not have someone to talk to. This could contribute to an increase in AI-related anxiety. Higher educated people may also have a more nuanced view of the potential advantages and disadvantages of AI than lower educated people [10]. Concerning employment status, the comparatively high value for retired people is particularly striking. This could be explained by the fact that retired people are generally older and possibly less AI-savvy on average than younger people, which could encourage a certain amount of AI-related anxiety [47].

Interestingly, there are no AIAS-G differences with regard to migration background. However, it must at least be mentioned that the questionnaire was only available in German due to the validation in German. In this respect, it would be interesting to investigate possible differences between people without a migration background and people with a migration background and no knowledge of German.

An excellent reliability (Cronbach’s alpha of .97; learning = .98, job replacement = .94, sociotechnical blindness = .94, AI configuration = .95) of the AIAS-G was demonstrated in this study. The original validation study [5] also demonstrated an excellent Cronbach’s alpha of .96 (Cronbach’s alpha for the four factors: learning = .97, job replacement = .92, sociotechnical blindness = .92, AI configuration = .96). Similar results were found for the Turkish and Persian version [6,17].

Moderate (depressive and anxiety symptoms) to small correlations (life satisfaction and ikigai) were demonstrated with AI-related anxiety. Given the fact that AI-related anxiety cannot be equated with mental health outcomes such as depressive symptoms or anxiety symptoms, but rather represents a specific construct on its own (incorporating components such as job placement and sociotechnical blindness which are unique to the AI-related anxiety construct [5]), these results are very plausible. The strength of the association between AI-related anxiety and anxiety symptoms is comparable to other associations between specific anxieties and general anxiety symptoms. For example, a previous study found an association of climate anxiety and anxiety symptoms of r = .31, p < .001 [48]. Other specific anxieties, in contrast, were much more strongly associated with general anxiety symptoms (e.g., Coronavirus Pandemic Anxiety Scale, r = .76, p < .001, Fear of War Scale, r = .64, p < .001 [49,50]). It is not possible to compare such correlations with other validation studies of the AIAS because past studies did not report such associations.

The validation of the AIAS-G can facilitate future comparisons across different countries. This may enhance our present understanding of AI-related anxiety levels. Given the fact that existing studies used rather small, selective samples, which are not generalizable to entire populations, upcoming research based on representative samples of different countries would be desirable. We hope that the very favorable psychometric properties of the AIAS-G may inspire other researchers to use it in future research. For instance, potential positive and negative consequences of AI-related anxiety among populations speaking German could be investigated in future research.

We would like to highlight some strengths and limitations of our present study. First, a large, quota-based sample was used (representative in terms of age x sex, and federal state). Following recommendations, a carefully conducted translation process was performed. This is the first validation study of the German version of the AIAS. Our study employed a cross-sectional design. Consequently, it was impossible to assess a test-retest reliability or responsiveness. Our sample was restricted to individuals aged 18–74 years. Upcoming research is needed to quantify AI-related anxiety amongst adolescents as well as among individuals in old age. It would also be interesting to investigate the AI-related anxiety specifically in industries that are particularly affected by AI progress. It is worth noting that individuals fearing technological developments may be somewhat underrepresented in such online panels. This should be kept in mind when interpreting our present findings. The mean values determined in this study for the AIAS-G could therefore also be rather conservative and presumably even higher in reality. Another limitation is that it is unclear to which specific technologies the AI anxiety scale refers (e.g., algorithm anxiety, machine learning anxiety, or deep learning anxiety). Moreover, cut-offs are currently missing for the AIAS (e.g., for individuals with a high AI anxiety). A further limitation is that there are currently no recommendations for the AIAS on how to handle item nonresponse.

## 5 Conclusion

It can be concluded that the AIAS-G is a psychometrically sound instrument designed to determine AI anxiety levels among German speakers. Further translation and validation studies are needed to enable cross-country comparisons. Moreover, the test-retest reliability or responsiveness should be calculated in future longitudinal studies. Furthermore, future studies should clarify the predictors and consequences of AI anxiety in individuals speaking German.

## Supporting information

S1 Table Comparison of the target quote (according to EUROSTAT 2021) and our sample (in terms of sex x age). (DOCX)

S2 Table Comparison of the target quote (according to EUROSTAT 2021) and our sample (in terms of federal state). (DOCX)

S3 Table Results of the post-hoc tests (for Table 2 of the manuscript). (DOCX)

S4 Fig Histogram (scores of the subscales). (TIF)

S5 Table Description (items). (DOCX)
