# Supplementary material for: Translation and validation of the artificial intelligence anxiety scale in German
Source: PLoS One. 2025 Oct 8;20(10):e0333073. doi: 10.1371/journal.pone.0333073 (PMC12507318; doi:10.1371/journal.pone.0333073)
Supplement: S1 Table — (DOCX) [file pone.0333073.s001.docx]

S1 Table. Comparison of the target quote (according to EUROSTAT 2021) and our sample (in terms of sex x age)

|  | **Target quote** | | **Our sample** | |
| --- | --- | --- | --- | --- |
| Age group | Female | Male | Female | Male |
| 18 – 29 years | 9% | 10% | 9% | 9% |
| 30 – 39 years | 9% | 9% | 9% | 9% |
| 40 – 49 years | 8% | 8% | 8% | 8% |
| 50 – 59 years | 11% | 11% | 11% | 11% |
| 60 – 74 years | 13% | 12% | 13% | 12% |
