# Supplementary material for: Translation and validation of the artificial intelligence anxiety scale in German
Source: PLoS One. 2025 Oct 8;20(10):e0333073. doi: 10.1371/journal.pone.0333073 (PMC12507318; doi:10.1371/journal.pone.0333073)
Supplement: S2 Table — (DOCX) [file pone.0333073.s002.docx]

S2 Table. Comparison of the target quote (according to EUROSTAT 2021) and our sample (in terms of federal state)

|  | Target quote | Our sample |
| --- | --- | --- |
| Federal state |  |  |
| Baden-Wuerttemberg | 13% | 13% |
| Bavaria | 16% | 16% |
| Berlin | 4% | 5% |
| Brandenburg | 3% | 3% |
| Bremen | 1% | 1% |
| Hamburg | 2% | 2% |
| Hesse | 8% | 8% |
| Mecklenburg-Western Pomerania | 2% | 2% |
| Lower Saxony | 10% | 9% |
| North Rhine-Westphalia | 22% | 22% |
| Rhineland-Palatinate | 5% | 5% |
| Saarland | 1% | 1% |
| Saxony | 5% | 5% |
| Saxony-Anhalt | 3% | 3% |
| Schleswig-Holstein | 3% | 3% |
| Thuringia | 2% | 3% |
