# Supplementary material for: Translation and validation of the artificial intelligence anxiety scale in German
Source: PLoS One. 2025 Oct 8;20(10):e0333073. doi: 10.1371/journal.pone.0333073 (PMC12507318; doi:10.1371/journal.pone.0333073)
Supplement: S3 Table — (DOCX) [file pone.0333073.s003.docx]

S3 Table. **Results of the post-hoc tests (for Table 2 of the manuscript)**

Tukey-Kramer pairwise comparisons for variable sex

studentized range critical value(.05, 3, 3267) = 3.3160326

mean

grp vs grp group means dif TK-test

-------------------------------------------------------

1 vs 2 65.5192 73.5968 8.0776 10.0801*

1 vs 3 65.5192 66.7778 1.2586 0.1646

2 vs 3 73.5968 66.7778 6.8191 0.8917

Notes:

* < .05

Group 1 = male

Group 2 = female

Group 3 = diverse

Tukey-Kramer pairwise comparisons for variable age group

studentized range critical value(.05, 5, 3265) = 3.8598518

mean

grp vs grp group means dif TK-test

-------------------------------------------------------

1 vs 2 70.0773 66.5847 3.4926 2.6122

1 vs 3 70.0773 66.4810 3.5963 2.6330

1 vs 4 70.0773 70.6907 0.6134 0.4786

1 vs 5 70.0773 72.6133 2.5360 2.0301

2 vs 3 66.5847 66.4810 0.1037 0.0765

2 vs 4 66.5847 70.6907 4.1060 3.2335

2 vs 5 66.5847 72.6133 6.0286 4.8735*

3 vs 4 66.4810 70.6907 4.2097 3.2379

3 vs 5 66.4810 72.6133 6.1323 4.8358*

4 vs 5 70.6907 72.6133 1.9226 1.6336

Notes:

* < .05

Group 1 = 18 to 29 years

Group 2 = 30 to 39 years

Group 3 = 40 to 49 years

Group 4 = 50 to 59 years

Group 5 = 60 years and older

Tukey-Kramer pairwise comparisons for variable marital status

studentized range critical value(.05, 5, 3265) = 3.8598518

mean

grp vs grp group means dif TK-test

-------------------------------------------------------

1 vs 2 68.6399 76.9495 8.3096 5.2664*

1 vs 3 68.6399 76.7087 8.0689 3.3760

1 vs 4 68.6399 68.6802 0.0404 0.0434

1 vs 5 68.6399 67.7913 0.8486 0.3729

2 vs 3 76.9495 76.7087 0.2407 0.0907

2 vs 4 76.9495 68.6802 8.2692 5.5869*

2 vs 5 76.9495 67.7913 9.1582 3.5914

3 vs 4 76.7087 68.6802 8.0285 3.4506

3 vs 5 76.7087 67.7913 8.9174 2.8595

4 vs 5 68.6802 67.7913 0.8889 0.4025

Notes:

* < .05

Group 1 = single

Group 2 = divorced

Group 3 = widowed

Group 4 = living together: Married/Partnership

Group 5 = living separated: Married/Partnership

Tukey-Kramer pairwise comparisons for variable education

studentized range critical value(.05, 3, 3267) = 3.3160326

mean

grp vs grp group means dif TK-test

-------------------------------------------------------

1 vs 2 76.9407 71.6450 5.2957 3.8485*

1 vs 3 76.9407 65.4895 11.4512 8.2314*

2 vs 3 71.6450 65.4895 6.1555 7.2673*

Notes:

* < .05

Group 1 = low

Group 2 = medium

Group 3 = high

Tukey-Kramer pairwise comparisons for variable employment status

studentized range critical value(.05, 3, 3267) = 3.3160326

mean

grp vs grp group means dif TK-test

-------------------------------------------------------

1 vs 2 65.9233 75.0247 9.1014 8.5621*

1 vs 3 65.9233 72.0595 6.1362 6.6535*

2 vs 3 75.0247 72.0595 2.9652 2.5647

Notes:

* < .05

Group 1 = full-time employed

Group 2 = retired

Group 3 = other
