# Supplementary figures and images for: Translation and validation of the artificial intelligence anxiety scale in German
Source: PLoS One. 2025 Oct 8;20(10):e0333073. doi: 10.1371/journal.pone.0333073 (PMC12507318; doi:10.1371/journal.pone.0333073)

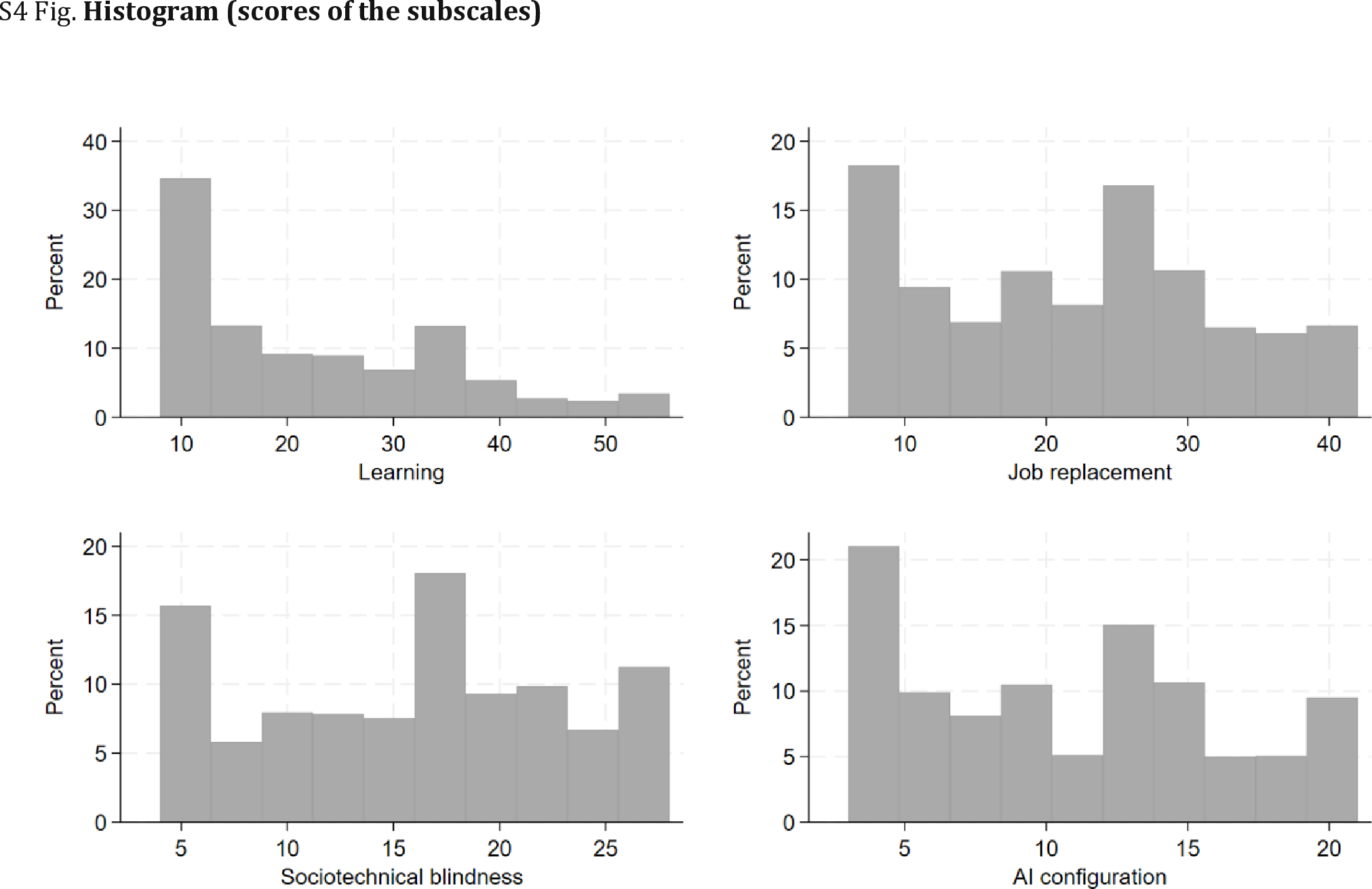

Supplement: S4 Fig — (TIF) [file pone.0333073.s004.tif]
