# Supplementary material for: Translation and validation of the artificial intelligence anxiety scale in German
Source: PLoS One. 2025 Oct 8;20(10):e0333073. doi: 10.1371/journal.pone.0333073 (PMC12507318; doi:10.1371/journal.pone.0333073)
Supplement: S5 Table — (DOCX) [file pone.0333073.s005.docx]

S5 Table. **Description (items)**

Missing N Response categories Alpha Loevinger Number of

data rate 1 2 3 4 5 6 7 - item Hj coeff NS Hjk

-----------------------------------------------------------------------------------------------------------------

Item 1 0.00% 3270 32.26% 16.06% 13.27% 19.72% 9.02% 3.94% 5.72% 0.97 0.83 0

Item 2 0.00% 3270 37.68% 18.10% 11.99% 16.36% 7.40% 3.67% 4.80% 0.97 0.87 0

Item 3 0.00% 3270 37.71% 17.92% 12.63% 16.27% 7.16% 3.73% 4.59% 0.97 0.88 0

Item 4 0.00% 3270 37.80% 18.01% 12.32% 16.33% 6.97% 3.82% 4.74% 0.97 0.88 0

Item 5 0.00% 3270 37.58% 17.68% 12.75% 16.48% 6.76% 4.07% 4.68% 0.97 0.87 0

Item 6 0.00% 3270 39.11% 16.94% 12.54% 15.23% 7.03% 4.28% 4.86% 0.97 0.83 0

Item 7 0.00% 3270 40.64% 16.97% 12.11% 14.92% 7.19% 3.85% 4.31% 0.97 0.84 0

Item 8 0.00% 3270 30.83% 16.06% 15.14% 17.61% 9.39% 5.23% 5.75% 0.98 0.78 0

-----------------------------------------------------------------------------------------------------------------

Item 9 0.00% 3270 24.50% 12.20% 13.46% 18.35% 11.93% 9.48% 10.09% 0.94 0.75 0

Item 10 0.00% 3270 23.24% 11.93% 12.32% 19.33% 13.12% 9.85% 10.21% 0.94 0.75 0

Item 11 0.00% 3270 21.87% 11.83% 12.97% 18.38% 13.06% 9.79% 12.11% 0.93 0.79 0

Item 12 0.00% 3270 20.76% 12.05% 13.79% 17.61% 13.61% 9.79% 12.39% 0.93 0.76 0

Item 13 0.00% 3270 26.57% 14.95% 12.78% 18.38% 12.66% 7.13% 7.52% 0.94 0.71 0

Item 14 0.00% 3270 19.60% 11.38% 13.09% 19.20% 13.18% 10.67% 12.87% 0.93 0.78 0

-----------------------------------------------------------------------------------------------------------------

Item 15 0.00% 3270 14.86% 7.52% 10.40% 17.34% 15.08% 12.72% 22.08% 0.93 0.79 0

Item 16 0.00% 3270 19.05% 10.89% 13.00% 20.43% 15.02% 10.37% 11.25% 0.92 0.82 0

Item 17 0.00% 3270 17.49% 10.98% 12.51% 18.32% 14.56% 11.65% 14.50% 0.90 0.85 0

Item 18 0.00% 3270 21.59% 12.51% 13.06% 19.11% 13.33% 8.75% 11.65% 0.93 0.80 0

-----------------------------------------------------------------------------------------------------------------

Item 19 0.00% 3270 20.86% 12.02% 12.94% 18.84% 13.24% 8.78% 13.33% 0.94 0.88 0

Item 20 0.00% 3270 22.97% 14.07% 13.94% 18.59% 13.12% 7.19% 10.12% 0.92 0.89 0

Item 21 0.00% 3270 25.75% 14.13% 14.31% 18.75% 10.58% 6.12% 10.37% 0.92 0.89 0

-----------------------------------------------------------------------------------------------------------------
